# Supplementary material for: An affinity-matured human monoclonal antibody targeting fusion loop epitope of dengue virus with in vivo therapeutic potency
Source: Sci Rep. 2021 Jun 21;11:12987. doi: 10.1038/s41598-021-92403-9 (PMC8217507; doi:10.1038/s41598-021-92403-9)
Supplement: Supplementary file 1 — Supplementary Information. [file 41598_2021_92403_MOESM1_ESM.docx]

**Supplementary information**

**An affinity-matured human monoclonal antibody targeting fusion loop epitope of dengue virus with *in vivo* therapeutic potency**

Tomohiro Kotaki^a,b†*^, Takeshi Kurosu^c^, Ariadna Grinyo-Escuer^d^, Edgar Davidson^d^, Siti Churrotin^b^, Tamaki Okabayashi^e^, Orapim Puiprom^e^, Kris Cahyo Mulyatno^b^, Teguh Hari Sucipto^b^, Benjamin J. Doranz^d^, Ken-ichiro Ono^f^, Soegeng Soegijanto^b^, Masanori Kameoka^a*^

^a^Department of Public Health, Kobe University Graduate School of Health Sciences, Japan

^b^Collaborative Research Center for Emerging and Re-emerging Infectious Diseases, Institute of Tropical Disease, Airlangga University, Indonesia

^c^Department of Virology I, National Institute of Infectious Diseases (NIID), Japan

^d^Integral Molecular, Inc., PA, USA

^e^Mahidol-Osaka Center for Infectious Diseases (MOCID), Faculty of Tropical Medicine, Mahidol University, Thailand

^f^Medical & Biological Laboratories Corporation, Ltd., Japan

**^†^Present address:**

Department of Virology, Research Institute for Microbial Diseases, Osaka University, Japan.

***Correspondence:**

Department of Virology, Research Institute for Microbial Diseases, Osaka University, Japan.

Tomohiro Kotaki ([tkotaki@biken.osaka-u.ac.jp](mailto:tkotaki@biken.osaka-u.ac.jp))

Department of Public Health, Kobe University Graduate School of Health Sciences, Japan.

Masanori Kameoka ([mkameoka@port.kobe-u.ac.jp](mailto:mkameoka@port.kobe-u.ac.jp))


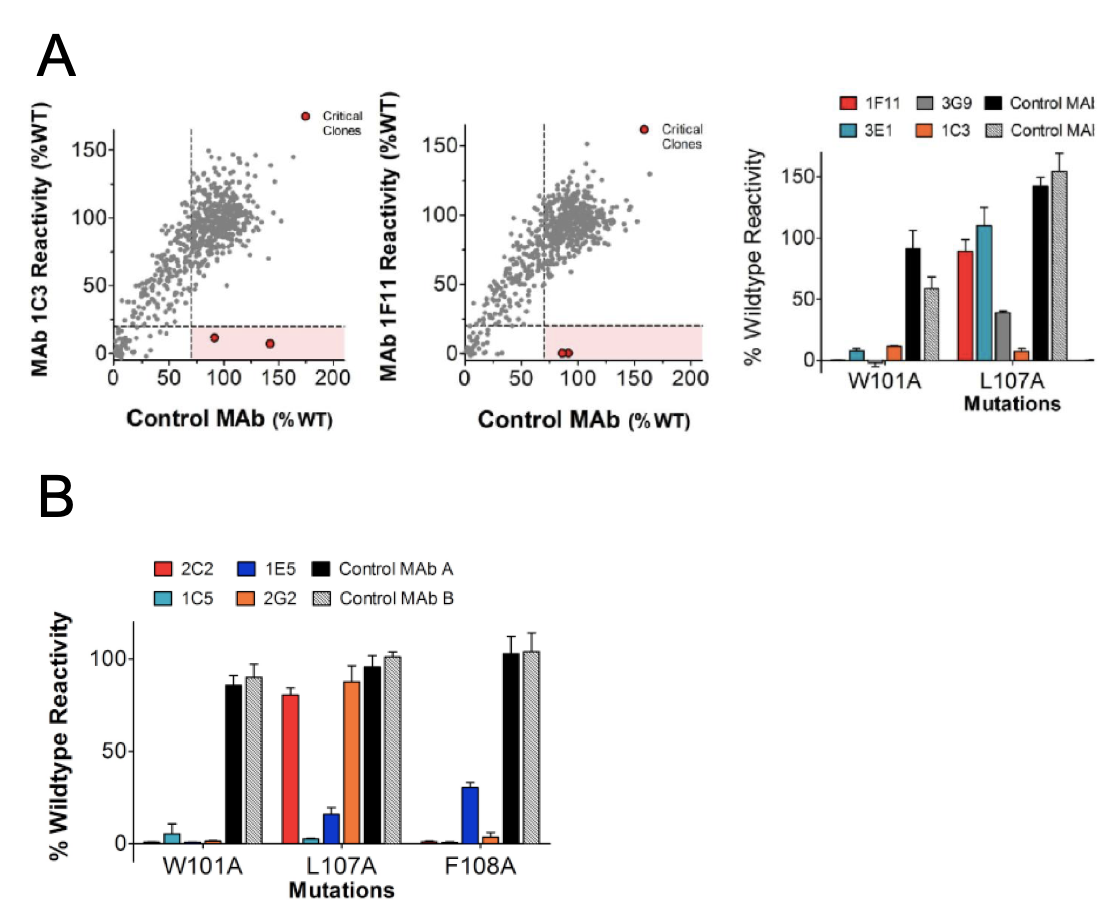


**Supplementary figure S1. Epitope mapping of the HuMAbs**

1. Identifying Critical Residues for MAbs 1C3, 1F11, 3E1, and 3G9. The DENV2 prM/E mutation library was assayed in an immunofluorescence flow cytometry assay, in duplicate, for binding by MAbs. Each raw data point was background-subtracted and normalized to the value for reactivity with wild-type DENV2 prM/E. We show graphs of 1C3 and 1F11 (representative of 1F11, 3E1, and 3G9) for the preliminary identification of critical clones. For each clone, the mean binding value is plotted as a function of the clone’s mean expression value (gray circles), given by binding to a control MAb. We applied binding thresholds (dashed lines) for the test MAb (<20% of reactivity with wild-type prM/E) and a control MAb (>70% of reactivity with wild-type) to identify critical clones (red circles).
2. Identifying Critical Residues for MAbs 2C2, 2G2, 1C5, and 1E5. The HuMAbs were screened on DENV2 prM/E mutants with an FLE mutation, along with two control MAbs that do not bind the fusion loop region. The average binding values for each clone are shown as a percentage of binding to wild-type DEN2 prM/E. Clones with reactivity <20% relative to wild-type prM/E were identified as critical for MAb binding.


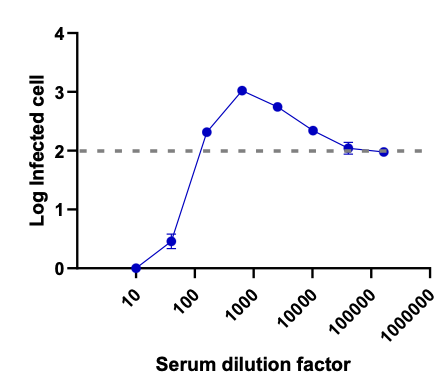


**Supplementary figure S2. ADE assay using DENV-2 NGC and DENV-2 immunized mouse serum**

Dotted line indicates the baseline of the infected cells in the control (100 infected cells; 2.0). Peak enhancement was observed at 1:640 diluted serum.

**Supplementaly table S1. Immunogenetic analysis of HuMAbs isolated in this study***

VH gene

|  | V gene | D gene | J gene | V region  AA mutations | CDR3 |
| --- | --- | --- | --- | --- | --- |
| 1C3 | IGHV3-72*01 | IGHD2-15*01 | IGHJ5*01 | 6.0%  (6/100) | ARDSGWYDF |
| 1E5 | IGHV3-30*03 | IGHD2-21*02 | IGHJ6*02 | 11.2%  (11/98) | ARGRPYIGGDYEYDYFYGMDV |
| 1F11 | IGHV3-30*04 | IGHD3-22*01 | IGHJ4*02 | 8.2%  (8/98) | AKDFLYYYDTNGDTGH |
| 2C2 | IGHV3-23*01 | IGHD6-13*01 | IGHJ4*02 | 9.2%  (9/98) | ATLFVAAPGTGY |
| 2G2 | IGHV3-23*01 | IGHD6-19*01 | IGHJ4*02 | 9.3%  (9/97) | AILFNSDSPLDY |
| 3E1 | IGHV3-23*01 | IGHD3-9*01 | IGHJ4*02 | 6.1%  (6/98) | VSLGDAWLLYWTP |
| 3G9 | IGHV3-23*02 | IGHD3-16*01 | IGHJ4*02 | 14.3%  (14/98) | AKLFGVGDSDGY |

VL gene

|  | V gene | J gene | V region  AA mutations | CDR3 |
| --- | --- | --- | --- | --- |
| 1C3 | IGLV2-14*01 | IGLJ3*02 | 16.3%  (16/98) | SSYTRSRTRV |
| 1E5 | IGKV2-24*01 | IGKJ2*03 | 4.0%  (4/99) | MQATHFPYS |
| 1F11 | IGLV7-46*01 | IGLJ7*01 | 3.1%  (3/98) | FLSYSGARPV |
| 2C2 | IGLV7-46*01 | IGLJ3*02 | 5.1%  (5/98) | LLSYSGGRPV |
| 2G2 | IGLV7-46*01 | IGLJ7*01 | 3.1%  (3/98) | FLSYSGARPV |
| 3E1 | IGKV2-30*02 | IGKJ4*01 | 6.0%  (6/100) | MQATHLPPT |
| 3G9 | IGLV7-46*01 | IGLJ3*02 | 7.1%  (7/98) | LLSYGGGRPV |

*HuMAb 1E5 was not successfully sequenced.

**Supplementary table S2. Sequence data of HuMAbs isolated in this study**

>1E5 VH

CAGGTGCAGCTGGTGGAGTCTGGGGGAGGCGTGGTCCAGCCTGGGAAGTCCCTGAGAGTCTCCTGTGCAGCCTCTGGATTCATCTTCAGTAGCTATGGCATGCACTGGGTCCGCCAGGCTCCAGGCAAGGGGCTAGAGTGGGTGGCAGTTATATCATATGATGGACGTTATAAGTTCTATGCAGACTCTGTGAAGGGCCGATTCACCATCTCCAGAGACGATTCCAGGAGTACACTGTATCTGCAAATGAACAGCCTGAGAAGTGACGACACGGCTGTGTATTACTGTGCGAGAGGCCGCCCTTATATAGGTGGTGACTACGAGTACGACTACTTCTACGGTATGGACGTCTGGGGCCAAGGGACCACGGTCATCGTCTCCTCA

>2C2 VH

GAGGTGCAGCTGTTGGAGTCTGGGGGAGGCTTGGAACAGCGGGGGGGGTCCCTGAGACTCTCCTGTGCAGCCTCTGGATTCACTTTTAGCGACTATGCCATGACCTGGGTCCGCCAGGCTCCAGGGAAGGGGCTGGAGTGGGTCTCAACTATTAGTGGTAGTGGTGGTGGCACATACTATGCAGACTCCGTTAAGGGCCGGTTCACCATCTCCAGAGACAATTCCCAGAACACGCTGTATCTGCAAATGAACAGCCTGAGAGCCGAGGACACGGCCATATATTACTGTGCAACTTTATTCGTTGCAGCACCTGGGACGGGGTACTGGGGCCAGGGAACCCTGGTCACCGTCTCCTCA

>2G2 VH

GAGGTGCAGCTGTTGGAGTCTGGGGGAGGCGTGGTACAGCCGGGGGGGTCCCTGAGACTCTCCTGTGCAGCCTCTGGATTCACCTTTAGCAGCTATGCCATGACCTGGGTCCGCCAGGCTCCAGGGAAGGGGCTGGAGTGGGTCTCAACTATTGGTGGTAGTGGTGATACATACTACTCAGACTCCGTGATGGGCCGGTTCACCATCTCCAGAGACAATTCCAAGAATACGCTGTATCTGCAAATGAACAGCCTGAGAGTCGAGGACACGGCCGTATATTACTGTGCCATTTTGTTTAATAGTGACTCTCCCCTCGACTACTGGGGCCAGGGAACCCTGGCCACCGTCTCCGCA

>3E1 VH

GAGGTGCAGCTCTTGGAGTCTGGGGGAGGCTTGGTACAGCCTGGGGGGTCCCTGAGACTCTCCTGTGCAGCCTCTGGATTCACCTTTAGCAGATTTGCCATGACCTGGGTCCGCCAGGCTCCAGGGAAGGGGCTGGAGTGGGTCTCGGCTATTAGTGGGAGTGGTGGTAGTACGTTCTACGCAGACTCCGTGAAGGGCCGGTTCACCATCTCCAGAGACAATTCCAAGAACACGCTGTATCTGCAAATGAACAGCCTGAGAGCCGAGGACACGGCCGTATATTATTGTGTCTCCCTAGGAGATGCCTGGTTATTATATTGGACTCCGTGGGGCCAGGGAAGCCTGGTCACCGTCTCCTCA

>3G9 VH

GAGGTGCAGCTGTTGGACTCTGGGGGAGGCTTGGTGCAGCCTGGGGGGTCCCTGAGACTCTCCTGTGCAGCCTCTGGATTCACCTTTAACGACTATGCCATGACCTGGGTCCGCCAGGCTCCGGGGAAGGGGCTGGAGTGGGTCTCGACTATTAGTGGTAGTGGTGATGGCACTTACTACCAAGAGTCCCTGAAGGGCCGGTTCACCATCTCAAGAGACAATTCCAAGAATACGTTGTATCTACTAATGAGCAGCCTGAGAGTCGACGACACGGCCGTCTATTACTGTGCGAAACTATTCGGTGTCGGGGACTCGGATGGGTACTGGGGCCAGGGAACCCTGGTCACCGTCTCCTCA

>1C3 VH

GAGGTGCAGCTGGTGGAGTCTGGGGGAGGCTTGGTCCAGCCTGGAGGGTCCCTGAGACTCTCCTGTGCAGCCTCTGGATTCACCTTCAGTGACCACTACATAGACTGGGTCCGCCAGGCTCCAGGGAAGGGGCTGGAGTGGGTTGGCCGTACTAGCAACAGAGCTAACTCTTACACCTCAGAATACGCCGCGTCTGTGAAAGGCAGATTCACCATCTCAAGAGATGATTCAAAGAACTCAGTGTATCTGCAAATGAACAGCCTGAAAACCGAGGACACGGCCGTGTATTTCTGTGCTAGAGATAGTGGTTGGTACGACTTCTGGGGCCAGGGAACCCTGGTCACCGTCTCCTCA

>1F11 VH

CAGGTGCAGCTGGTGGAGTCTGGGGGAGGCGTGGTCCAGCCTGGGAAGTCCCTGAGACTCTCCTGTGCAGCCTCTGGATTCACGTTCGAAAACTATGCCATGCACTGGGTCCGCCAGGCTCCAGGCAAGGGGCTGGAGTGGGTGGCAGTTATATCGTATGTTGAAAGTGAGAAATACTACGCAGACTCCGTGAAGGGCCGATTCACCATCTCCAGAGACAATTCCAAGAACACGCTGGATCTGCAAATGAACAGCCTGAGAGCTGAGGACACGGCTGTGTATTACTGTGCGAAAGATTTTCTCTATTACTATGATACTAATGGTGACACTGGCCACTGGGGCCAGGGAACCCTGGTCACCGTCTCTTCA

>1C3 VL

TCTGCCCTGACTCAGCCTGCCTCCGTGTCTGGGTCTCCTGGACAGTCGATCACCATCTCCTGCACTGGACCCAGCAGAGACCTTGGTGCTAATAACTATGTCTCCTGGTACCAGCAGCACCCAGGCAAAGCCCCCAAACTCTTGATTTATGATATCAATAATCGGCCCTCAGGGGTCCCTGATCGCTTCTCTGGGTCCAGGTCTGGCAACACGGCCTCCCTGACCATCTCTGGGCTCCAGGCTGACGACGAGGCTGATTATTACTGCAGCTCATATACACGCAGCAGAACTAGGGTGTTCGGCGGAGGGACCAAGCTGACCGTCCTA

>1E5 VL

ATTGTGATGACCCAGACTCCACTGTCCTCACCTGTCACCCTTGGACAGCCGGCCTCCATCTCCTGCAGGTCTAGTCAAAGCCTCGTACACAGTGATGGAAACACCTACTTAAATTGGCTTCAGCAGAGGCCAGGCCAGCCTCCAAGACTCCTGATTTATAACATTTCTAACCGGTTCTCTGGGGTCCCAGACAGATTCAGTGGCAGTGGGGCAGGGACAGATTTCACACTGAAAATCAACAGGGTGGAAGCTGAGGATGTCGGGGTTTATTACTGCATGCAAGCTACACATTTTCCGTACAGTTTTGGCCAGGGGACCAAGCTGGAGATCAAA

>1F11 VL

CAGGCTGTGGTGACTCAGGAGCCCTCACTGACTGTGTCCCCAGGAGGGACAGTCACTCTCACCTGTGGCTCCAGTACTGGAGCTGTCACCAGTGGTCATTTTCCCTACTGGTTCCAGCAGAAGCCTGGCCAAGCCCCCAGGACACTGATTTATCATACAAGCAACAAACACTCCTGGACACCTGCCCGGTTCTCAGGCTCCCTCCTTGGGGGCAAAGCTGCCCTGACCCTTTCGGGTGCGCAGCCTGAGGATGAGGCTGAGTATTACTGCTTCCTCTCCTATAGTGGTGCTCGGCCTGTGTTCGGAGGAGGCACCCAACTGACCGTCCTC

>2C2 VL

CAGGCTGTGGTGACTCAGGAGCCCTCACTGACTGTGTCCCCAGGAGGGACAGTCACTCTCACCTGTGTCTCCAGCACTGGAGCTGTCACCAGTGGTCATTATCCCTACTGGTTCCAGCAGAAGCCTGGCCAAGCCCCCAGGACACTGATTTATCATACAAGCAACAAACACTCCTGGACACCTGCCCGGTTCTCAGGCTCCGTCCTTGGGGGCAAAGCTGCCCTGACCCTTTCGGGTGCGCAGCCTGAGGATGAGGCTGACTATTACTGCTTGCTCTCCTATAGTGGTGGTCGGCCGGTGTTCGGCGGAGGGACCAAGCTGACCGTCCTA

>2G2 VL

CAGGCTGTGGTGACTCAGGAGCCCTCACTGACTGTGTCCCCAGGAGGGACAGTCACTCTCACCTGTGGCTCCAGTACTGGAGCTGTCACCAGTGGTCATTTTCCCTACTGGTTCCAGCAGAAGCCTGGCCAAGCCCCCAGGACACTGATTTATCATACAAGCAACAAACACTCCTGGACACCTGCCCGGTTCTCAGGCTCCCTCCTTGGGGGCAAAGCTGCCCTGACCCTTTCGGGTGCGCAGCCTGAGGATGAGGCTGAGTATTACTGCTTCCTCTCCTATAGTGGTGCTCGGCCTGTGTTCGGAGGAGGCACCCAACTGACCGTCCTC

>3E1 VL

GATGTTGTGATGACTCAGTCTCCACTCTCCCTGCCCGTCACCCTTGGACAGCCGGCCTCCATCTCCTGCAGGTCTAGTCAAAGCCTCGTACACGGTGATGGAAACACCTACTTGAATTGGTTTCAGCAGAGGCCAGGCCAATCTCCAAGGCGCCTAATTTATAACGTTTCTAACCGGGACCCTGGGGTCCCAGACAGATTCAGCGGCAGTGGGTCAGGCACTGATTTCACACTGAAAATCAGCTGGGTGGAGGCTGAGGATGTTGGGGTTTACTACTGCATGCAAGCTACACACTTGCCCCCAACTTTCGGCGGAGGGACCAAGGTGGAGATC

>3G9 VL

CAGGCTGTGGTGACTCAGGAGCCCTCACTGACTGTGTCCCCAGGAGGGACAGTCACTCTCACCTGTGGCTCCAGCACTGGAGCTGTCACCAGTGATCACTATTTCTACTGGTTCCAGCAGAAGCCTGGCCAAGCCCCCAGGACACTGATTTATCAGACAACCAACAAACATCCCTGGACACCTGCCCGGTTCTCAGGCTCCCTCCTTGGGGGCAAGGCAGCCCTGACCCTTTCGGGTGCGCAGCCTGAGGATGAGGCTGAGTATTATTGTTTGCTCTCCTATGGTGGTGGTCGGCCGGTGTTCGGCGGAGGGACCAAGCTGACCGTCCTA
